# Sitagliptin After Ischemic Stroke in Type 2 Diabetic Patients: A Nationwide Cohort Study

**DOI:** 10.1097/MD.0000000000001128

**Published:** 2015-07-17

**Authors:** Dong-Yi Chen, Szu-Heng Wang, Chun-Tai Mao, Ming-Lung Tsai, Yu-Sheng Lin, Feng-Chieh Su, Chung-Chuan Chou, Ming-Shien Wen, Chun-Chieh Wang, I-Chang Hsieh, Kuo-Chun Hung, Wen-Jin Cherng, Tien-Hsing Chen

**Affiliations:** From the Division of Cardiology (DYC, MLT, CCC, MSW, CCW, ICH, KCH), Department of Internal Medicine; Department of Medical Education (SHW), Chang Gung Memorial Hospital, Chang Gung University College of Medicine; Division of Cardiology (CTM, WJC, THC), Department of Internal Medicine, Chang Gung Memorial Hospital, Keelung, and Chang Gung University College of Medicine, Taoyuan; Division of Cardiology (YSL), Department of Internal Medicine, Chang Gung Memorial Hospital, Chiayi, and Chang Gung University College of Medicine, Taoyuan; and Department of Neurology (FCS), Chang Gung Memorial Hospital, Keelung, Taiwan.

## Abstract

The cerebrovascular safety and efficacy of sitagliptin, a dipeptidyl peptidase-4 inhibitor, in patients with type 2 diabetes mellitus (T2DM) with ischemic stroke remains uncertain. The aim of this study was to assess the efficacy and safety of sitagliptin in patients with T2DM with recent ischemic stroke.

We analyzed data from the Taiwan National Health Insurance Research Database between March 1, 2009, and December 31, 2011. Ischemic stroke patients were identified from individuals with T2DM. Patients who received sitagliptin were compared with those who did not to evaluate the cardiovascular safety and efficacy of sitagliptin. The primary outcome was a composite of ischemic stroke, myocardial infarction, or cardiovascular death.

A total of 5145 type 2 diabetic patients with ischemic stroke met our inclusion criteria and were followed for up to 2.83 years (mean, 1.17 years). Overall, 1715 patients (33.3%) received sitagliptin and 3430 patients (66.7%) did not. The primary composite outcome occurred in 190 patients in the sitagliptin group (11.1%) and in 370 patients in the comparison group (10.8%) (hazard ratio [HR] = 1.02; 95% confidence interval [CI], 0.85–1.21). Patients treated with sitagliptin had a similar risk of ischemic stroke, hemorrhagic stroke, and all-cause mortality with an HR of 0.95 (95% CI, 0.78–1.16, *P* = 0.612), 1.07 (95% CI, 0.55–2.11, *P* = 0.834), and 1.00 (95% CI, 0.82–1.22, *P* = 0.989), respectively, compared with patients not treated with sitagliptin.

Treatment with sitagliptin in type 2 diabetic patients with recent ischemic stroke was not associated with increased or decreased risks of adverse cerebrovascular outcomes.

## Abstract 2

Supplemental Digital Content is available in the text

## INTRODUCTION

It is well established that type 2 diabetes mellitus (T2DM) is associated with an elevated risk of major cardiovascular complications.^1^ Patients with DM have a 2-fold excess risk for ischemic stroke compared with individuals without DM.^2^ From another perspective, acute stroke can cause stress hyperglycemia with increased mortality and poor outcome.^3^ Although improved glycemic control has been shown to reduce the risk of microvascular complications of T2DM, its benefit for macrovascular risk reduction has been controversial.^4–8^ There is still uncertainty regarding the cardiovascular outcome benefits or neuroprotective effect of glucose-lowering treatment after stroke.

Dipeptidyl peptidase-4 (DPP-4) inhibitors are used for the treatment of patients with T2DM. These agents enhance the availability of incretin hormones such as glucagon-like peptide 1 (GLP-1), which in turn stimulate insulin and suppress glucagon secretion.^9^ Emerging evidence has suggested that the DPP-4 inhibitor linagliptin may have neuroprotective effects and be associated with significantly fewer cardiovascular events and stroke.^10,11^ On the contrary, two recently published studies on saxagliptin and alogliptin did not find any beneficial effect on cardiovascular outcomes or stroke.^12,13^ As a result, there is ongoing debate about the cardiovascular benefits and potential risks of DPP-4 inhibitors.

Sitagliptin is the first approved DPP-4 inhibitor for clinical use. A meta-analysis study suggested that there is a decreased risk of adverse cardiovascular outcomes,^14^ but some individual studies reported a neutral effect,^15,16^ whereas others found increased cardiovascular risks.^17,18^ However, none of these studies designated ischemic stroke patients as the main study population. As a result, there are very limited data on the effects of sitagliptin in T2DM patients after ischemic stroke.

Given the current controversy over the neuroprotective effect of DPP-4 inhibitors, we conducted this nationwide cohort study to evaluate the efficacy and safety of sitagliptin with respect to cerebrovascular outcomes in patients with T2DM who had recent ischemic stroke.

## METHODS

### Data Source

We conducted a nationwide cohort study by using National Health Insurance Research Database (NHIRD) of Taiwan, which consists of standard computerized claim documents submitted by medical institutions through the National Health Insurance (NHI) program. The NHIRD has been described in the previous studies.^18^ Briefly, the NHI program covers the medical needs of >23 million people, who represent >99% of the population of Taiwan. All clinical diagnoses were recorded according to the International Classification of Diseases, Ninth Revision, Clinical Modification (ICD-9-CM) codes (Appendix, http://links.lww.com/MD/A330). The accuracy of diagnosis of major disease in the claims database, such as myocardial infarction (MI), stroke, and chronic kidney disease (CKD), has been validated.^19–21^ The information and records of patients were deidentified prior to analysis. This study was approved by the Ethics Institutional Review Board of Chang Gung Memorial Hospital, Taoyuan, Taiwan.

### Study Cohorts

**FIGURE 1:**
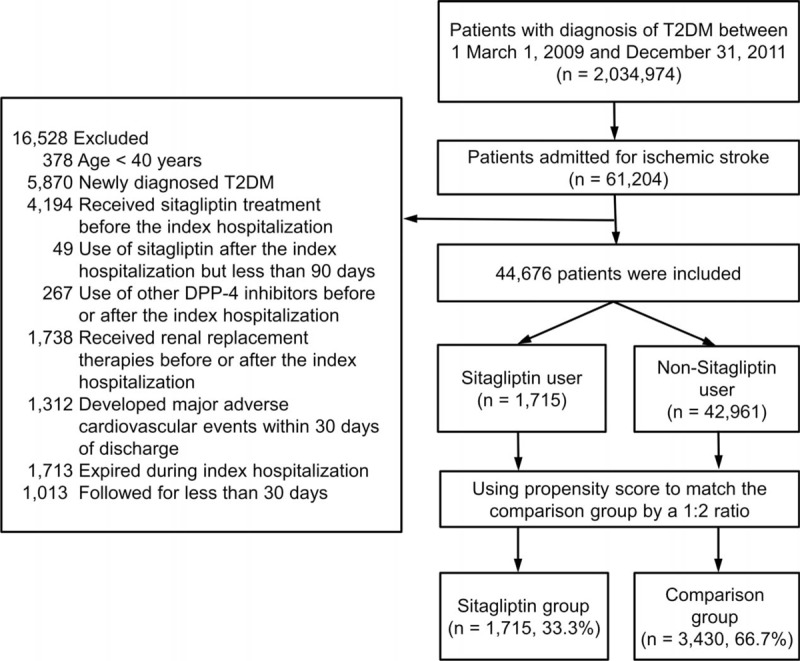
Enrolment of study patients. Patients with T2DM who were hospitalized for ischemic stroke were identified for our study cohort after relevant exclusions. DPP-4 = dipeptidyl peptidase-4, T2DM = type 2 diabetes mellitus.

We identified all patients in the NHIRD with T2DM (ICD-9-CM codes 250) between March 1, 2009, and December 31, 2011. Only patients with T2DM who were hospitalized for ischemic stroke (ICD-9-CM codes 433–435) were included in our study (Figure 1). The index hospitalization was defined as the date on which the patient was admitted for ischemic stroke. Patients’ baseline characteristics and comorbidities, including previous cerebrovascular accident and atrial fibrillation, and CHADS_2_ and CHA_2_DS_2_-VASc scores, were identified.^22,23^ The follow-up period was based on the index hospitalization to date of death, loss of follow-up, or until December 31, 2011.

### Exposure to Sitagliptin

Patients who received a prescription of sitagliptin for 90 consecutive days following index discharge were defined as the sitagliptin group, whereas patients who did not receive sitagliptin were defined as the comparison group. Sitagliptin dosages were prescribed according to Taiwan's NHI regulations: 100, 50, and 25 mg daily for patients with an estimated glomerular filtration rate of >50 mL/min, between 30 to 50, and <30 mL/min, respectively.

### Exclusion Criteria

Patients with age <40 years were initially excluded. Second, patients with newly diagnosed T2DM, which was defined as T2DM diagnosed during index hospitalization, were excluded to ensure consistencies in disease severity and duration among diabetic patients. In order to avoid carry-over effect, we excluded patients who received sitagliptin treatment before index hospitalization and those who were prescribed sitaglitpin for <90 days after index date. Patients who used DPP-4 inhibitors other than sitagliptin were also excluded. Third, patients were excluded if they met any of the following criteria that may affect the long-term outcomes: received renal replacement therapies before or after the index hospitalization; major adverse cardiovascular events (defined as ischemic stroke, MI, or cardiovascular death) within 30 days of discharge; expired during index admission; or followed up for <30 days after the index hospitalization.

### Study Outcomes and Covariate Measurements

The baseline comorbidities were identified by ICD-9-CM diagnosis codes and nonstudy medication use after the index hospitalization was also evaluated. The primary outcome was composite event of ischemic stroke, MI, or cardiovascular death. Definitions of cardiovascular death met the criteria of the Standardized Definitions for End Point Events in Cardiovascular Trials draft by the Food and Drug Administration.^24^ Death and causes of death were based on registry data of the NHIRD.^25^ Other secondary outcomes were hemorrhagic stroke, nonfatal ischemic stroke, nonfatal acute MI, deaths of any cause, and hospitalization for heart failure. Safety outcomes included acute or chronic pancreatitis, hypoglycemia, hyperosmolar hyperglycemic state, and diabetic ketoacidosis.

### Statistical Analysis

To minimize the bias in the estimated effect (group difference) in this study, the sitagliptin cohort was matched with the comparison cohort with a 1:2 ratio in terms of patient characteristics, baseline comorbidities, nonstudy medication prescribed 90 days since the index hospitalization (Table 1), and index year and month by using the propensity score method (PSM). The PSM matching algorithm was based on the nearest-neighbor method and used the caliper radius (set as 0.5 σ) that signifies a tolerance level for the maximum distance in the propensity score.^26^ The matching procedure was performed using SAS version 9.3 (SAS Institute, Cary, NC).

**TABLE 1:**
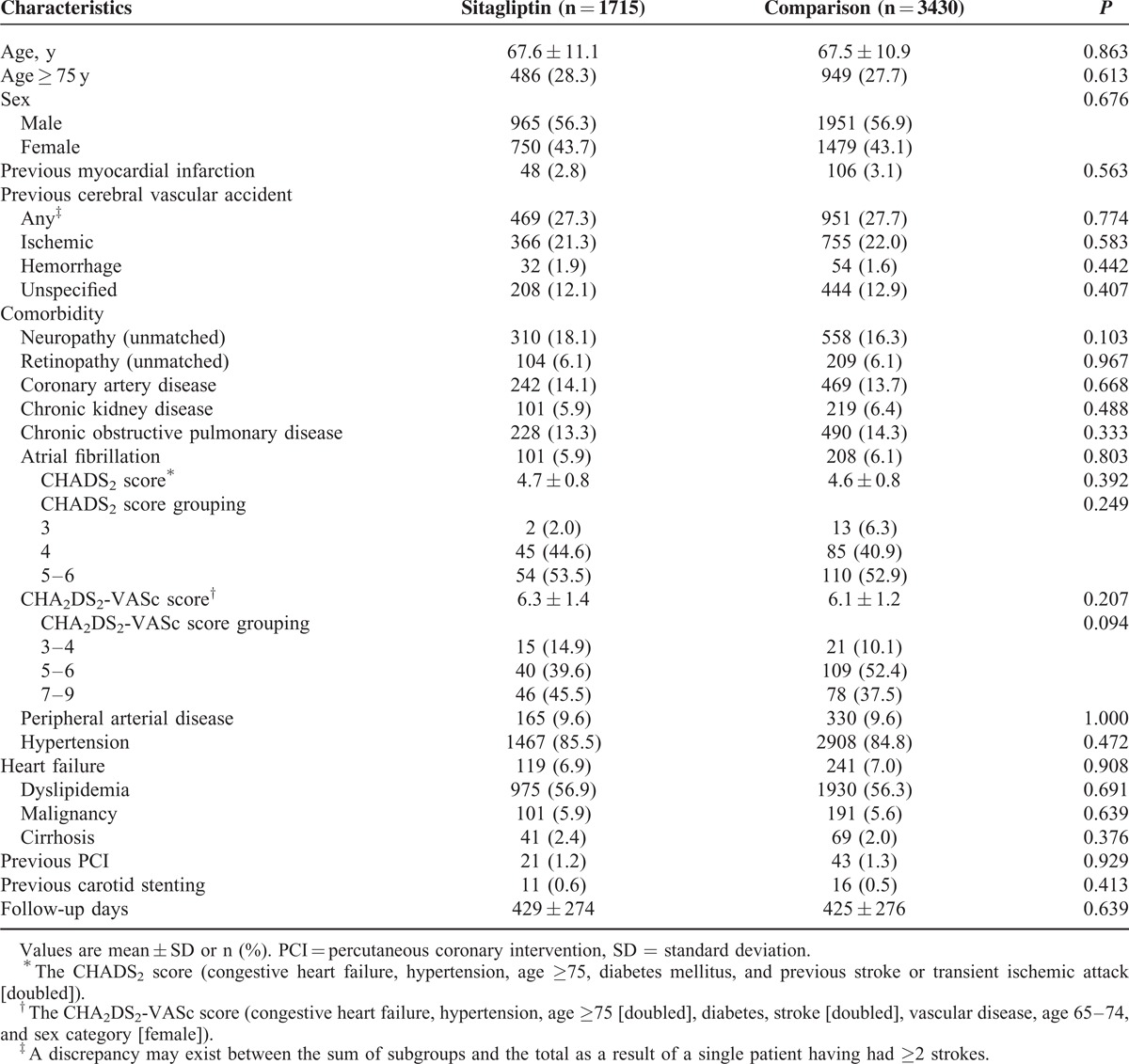
Baseline Demographic and Clinical Characteristics of the Study Patients

Clinical characteristics between study groups (sitagliptin and comparison groups) were compared by χ^2^ test for categorical variables and independent sample *t* test for continuous variables. Time to the first occurrence of a predefined primary or secondary outcome after the index hospitalization between the study groups was compared by Cox proportional hazard models with adjustment of the propensity score. The survival rates for the predefined period (ie, 3 months, 1 year, and until the last follow-up) for each study group were estimated and depicted by the Kaplan–Meier method, along with the log-rank test. All data analysis was conducted using IBM SPSS software version 22 (IBM SPSS Inc, Chicago, IL).

## RESULTS

### Study Patients

A total of 5145 patients diagnosed with T2DM who were hospitalized for ischemic stroke from March 1, 2009, through December 31, 2011, were identified for our study cohort. Of these patients, 1715 (33.3%) were in the sitagliptin group and 3430 matched patients (66.7%) were in the comparison group. The mean age for the overall cohort was 67.5 years (standard deviation [SD] = 11.0 years). The mean follow-up period was 1.17 years (SD = 0.75 years), and the maximum follow-up time was 2.83 years. No differences in the distribution of the baseline characteristics and comorbidities between the study groups were found after PSM matching (Table 1).

Patients with atrial fibrillation accounted for 5.9% of the patients in the sitagliptin group and 6.1% in the comparison group. The CHADS_2_ score was 4.7 for the sitagliptin group and 4.6 for the comparison group, and CHA_2_DS_2_-VASc score was 6.3 for the sitagliptin group and 6.1 for the comparison group. Atrial fibrillation, CHADS_2_ score, and CHA_2_DS_2_-VASc score were well matched between the 2 groups. The use of nonstudy medication for T2DM and cardiovascular disease after enrolment was also well balanced between both the groups (Table 2).

**TABLE 2:**
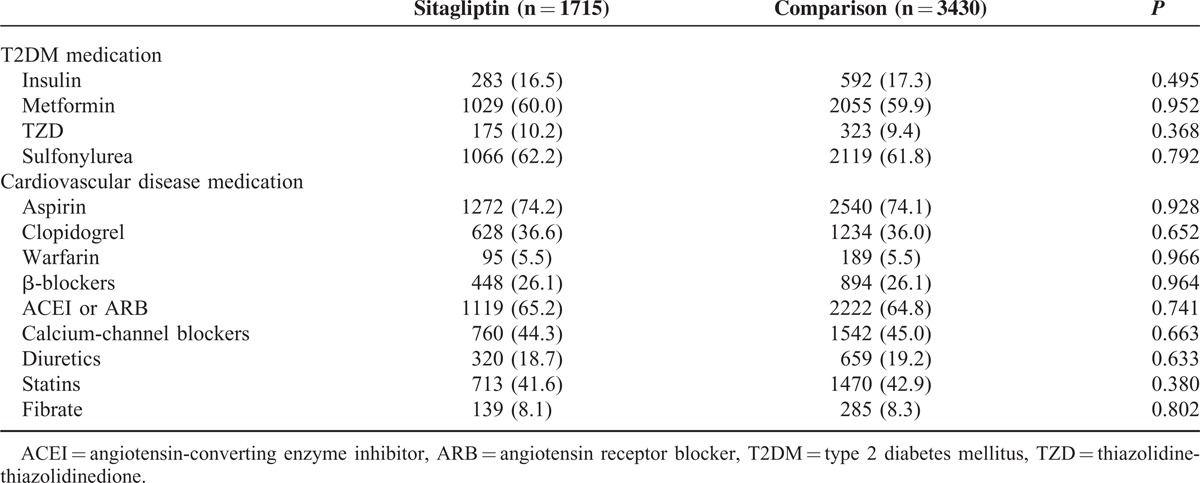
Proportions of Patients Receiving Nonstudy Medications

### Cardiovascular Outcomes

**FIGURE 2:**
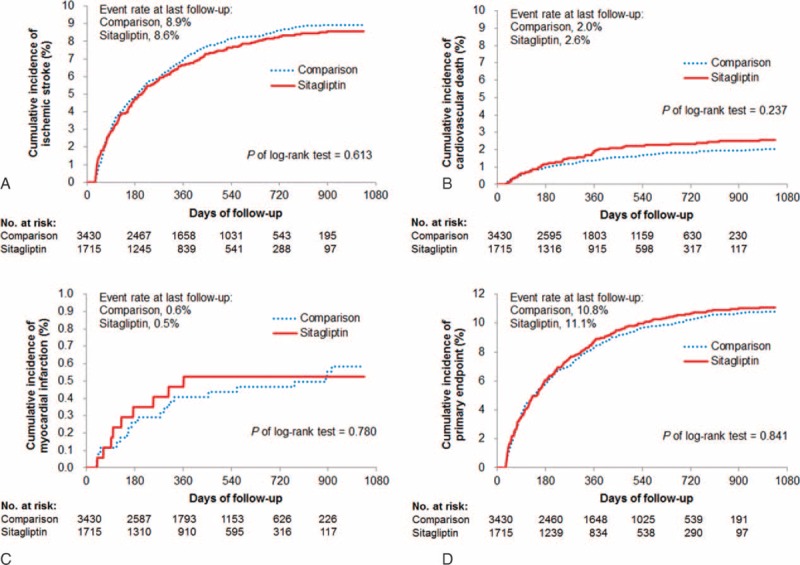
Cumulative probability of event rates in each study group for (A) ischemic stroke, (B) cardiovascular death, (C) myocardial infarction, and (D) primary composite endpoint. The primary endpoint was a composite of ischemic stroke, cardiovascular death, or myocardial infarction. No significant differences in the primary composite outcomes were observed between the 2 study groups after a mean 1.17-year follow-up.

The composite primary cardiovascular outcome occurred in 190 patients in the sitagliptin group (11.1%) and in 370 patients in the comparison group (10.8%) (hazard ratio [HR] = 1.02; 95% confidence interval [CI], 0.85–1.21, *P* = 0.845). The incidence rates of ischemic stroke (HR = 0.95; 95% CI, 0.78–1.16, *P* = 0.612), MI (HR = 0.90; 95% CI, 0.41–1.97, *P* = 0.785), and cardiovascular death (HR = 1.25; CI, 0.86–1.83, *P* = 0.243) were similar for the 2 study groups at 3-month follow-up and until the end of the study (Table 3; Figure 2A–D).

**TABLE 3:**
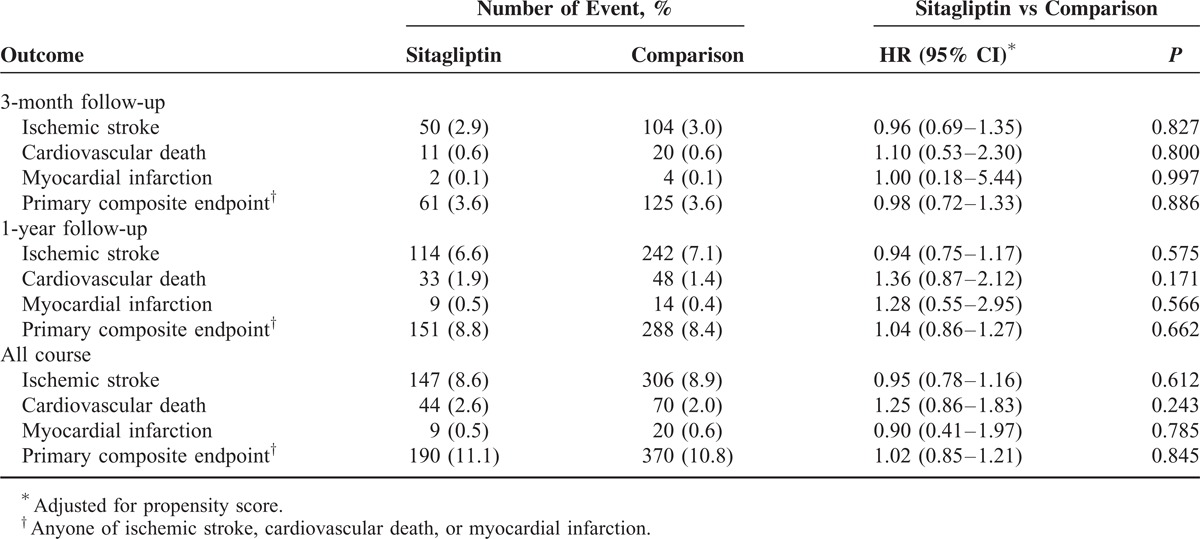
Primary Outcomes in Various Follow-Up Periods

**FIGURE 3:**
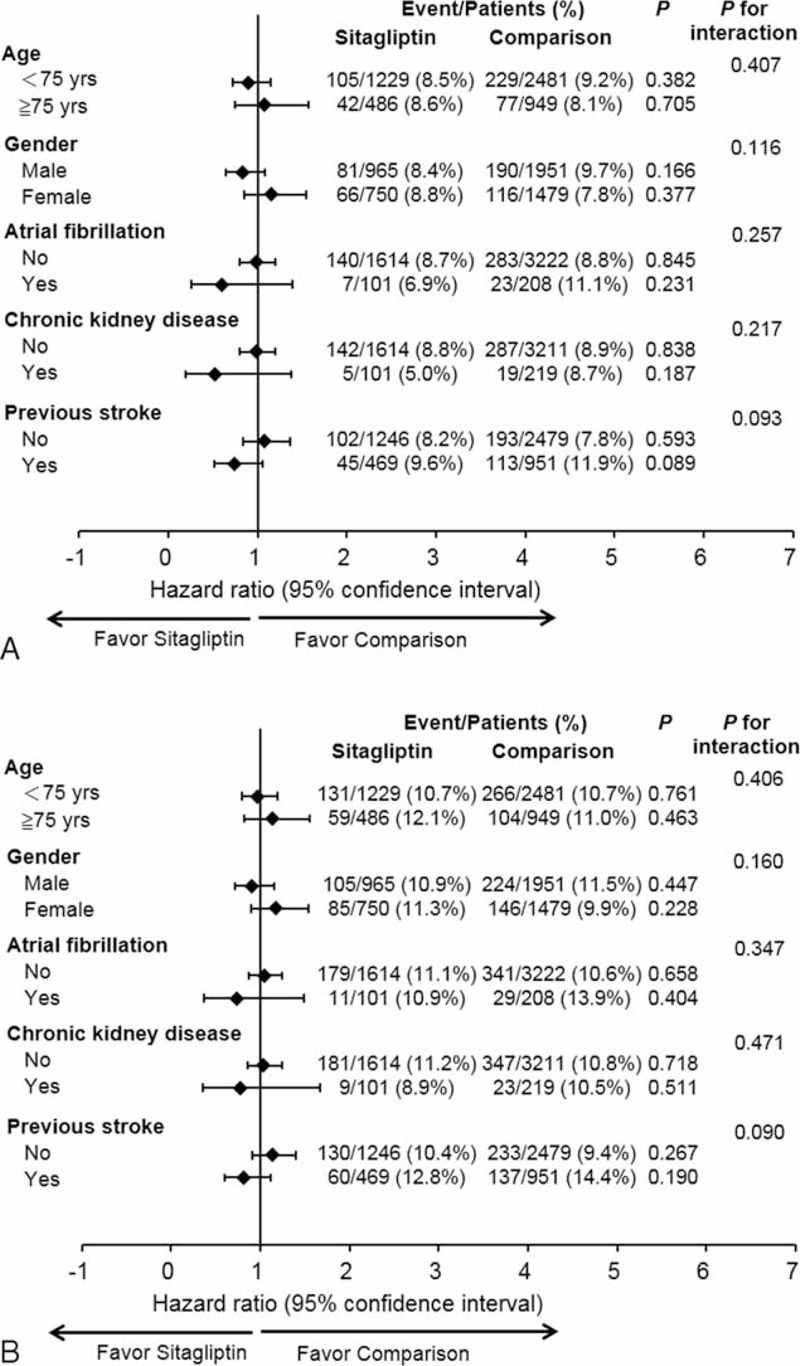
Subgroup analysis for (A) ischemic stroke and (B) primary composite endpoint. Sitagliptin use was associated with a neutral effect on ischemic stroke and primary composite outcomes in patients with or without a previous history of atrial fibrillation, chronic kidney disease, or cerebrovascular accident.

With regard to the secondary outcomes, there were no significant differences in the risks of hemorrhagic stroke (HR = 1.07; 95% CI, 0.55–2.11), nonfatal ischemic stroke (HR = 0.97; 95% CI, 0.79–1.18), nonfatal MI (HR = 1.12; 95% CI, 0.50–2.54), death of any cause (HR = 1.00; 95% CI, 0.82–1.22), or hospitalization for heart failure (HR = 0.79; 95% CI, 0.48–1.29) between the sitagliptin and comparison groups (Table 4). Subgroup analysis revealed that sitagliptin use was associated with a neutral effect on ischemic stroke or primary composite outcome in patients with or without previous history of atrial fibrillation, CKD, or cerebrovascular accident. There were no significant differences in adverse cardiovascular events between sexes, either (Figure 3A and B).

**TABLE 4:**
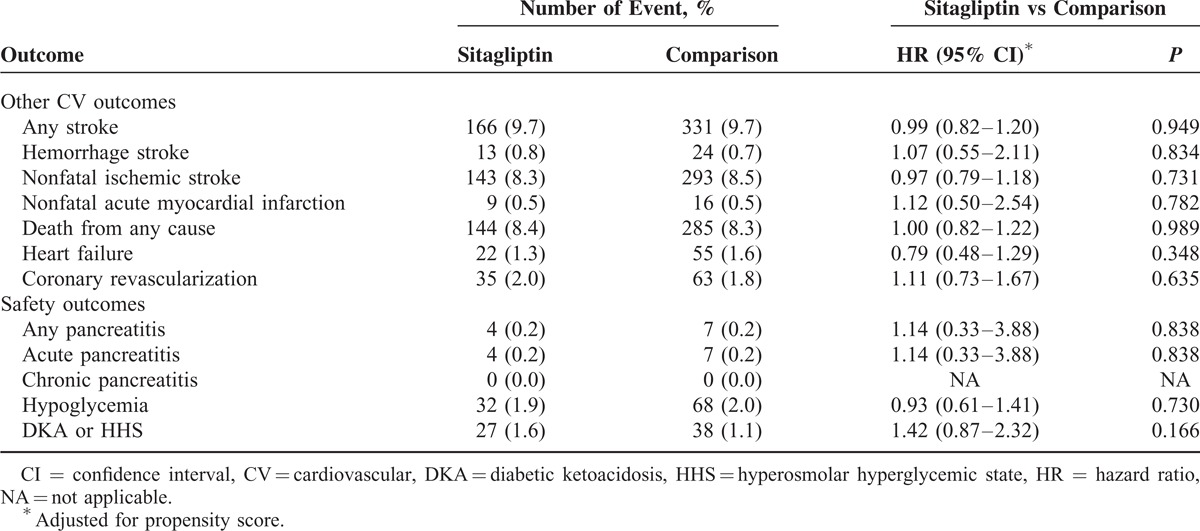
Secondary Outcomes

### Safety Outcomes

The sitagliptin and comparison groups did not differ significantly with respect to the incidence of hypoglycemia (1.9% and 2.0%; *P* = 0.730). The incidences of hyperosmolar hyperglycemic state and diabetic ketoacidosis were also similar across the 2 study groups (1.6% and 1.1%, respectively; *P* = 0.166). There was no significant difference in the incidence of acute or chronic pancreatitis between the 2 study groups (Table 4).

## DISCUSSION

This nationwide, population-based cohort study showed that in T2DM patients with recent ischemic stroke, treatment with the DPP-4 inhibitor sitagliptin neither significantly increased nor significantly reduced the major cardiovascular risks of ischemic stroke, MI, or cardiovascular death when compared with T2DM patients with ischemic stroke but not treated with sitagliptin at 3-month, 1-year, and all-course follow-up. Secondary outcome analysis demonstrated that the sitagliptin treatment group did not significantly differ from the comparison group with respect to risks of hemorrhagic stroke, nonfatal ischemic stroke, all-cause mortality, or hospitalization for heart failure. To our knowledge, this is the first study to evaluate the cardiovascular effect of sitagliptin focusing on the population of type 2 diabetic patients with recent ischemic stroke. Our results provide important information on the benefits and potential risks of using sitagliptin to treat this high-risk group of patients.

Our study revealed that the cerebrovascular outcomes in the sitagliptin treatment group were not inferior to those in the comparison group but did not provide a neuroprotective benefit for this high-risk group of patients. At present, there are still controversies about the neuroprotective effect of DPP-4 inhibitors. Sitagliptin treatment for 1 year has been shown to be associated with a beneficial effect with regard to the prevention of carotid intima-media thickness progression compared with the diet control.^27^ A 2-year study comparing linagliptin with glimepride in type 2 diabetic patients suggested that significantly fewer cardiovascular events and nonfatal stroke occur with linagliptin use.^10^ Nonetheless, the superiority of DPP-4 inhibition with regard to cardiovascular outcomes, such as cardiovascular death, ischemic stroke, and nonfatal stroke, was not found in 2 large cardiovascular outcome trials for saxagliptin in the SAVOR study^12^ and alogliptin in the EXAMINE study.^13^ More importantly, none of these studies designated ischemic stroke patients as the main study population. Uncertainty about the cerebrovascular benefits of different DPP-4 inhibitors among ischemic stroke patients remains.

Our goal was to determine the potential anti-stroke efficacy of sitagliptin, a DPP-4 inhibitor, for type 2 diabetic patients suffering from ischemic stroke. DPP-4 inhibitors do not cross the blood brain barrier (BBB), which inhibits their direct actions on the central nervous system. On the other hand, DPP-4 inhibitor-medicated increased GLP-1 can directly have an effect at the neuronal level in the brain which was suspected to be related the neuroprotective effect on the stroke mice.^28^ Furthermore, stroke-mediated damage has been reported to increase the permeability of the BBB but whether this effect could have benefit for neuroprotection of DPP-4 inhibitor is unclear. To this end, we included recent ischemic stroke type 2 diabetic patient receiving sitagliptin treatment and designed with cerebrovascular outcomes as the primary endpoints. However, our results didn’t find a significant anti-stroke efficacy mediated by sitagliptin treatment.

The exact mechanism underlying our finding that sitagliptin did not reduce the ischemic stroke rate remain unclear but there are several potential explanations. First, we included patients with a considerably high cardiovascular risk who had a recent episode of ischemic stroke, making the recurrent ischemic stroke rate more than 8% and primary composite cardiovascular event rate more than 10% during the mean follow-up period of 1.17 years. History of ischemic stroke is a strong predictor of recurrent stroke which may counterbalance the potential neuroprotective effect of sitaglipitin. In contrast, a 2-year study of linagliptin excluded patients with stroke or transient ischemic attack within 6 months before enrollment making their overall non-fatal stroke rate of only 0.9%.^10^ A difference in disease severity of the patient populations may have had an effect on the opposite conclusion reached by the two studies. Second, a majority of patients in our study received antiplatelet therapy with more than 74% of the patients receiving aspirin and more than 36% clopidogrel. The high proportion of aspirin use in our study was in accordance with 75% of patients receiving aspirin in the SAVOR study^12^ and 90% in the EXAMINE study.^13^ Both of these studies suggested a neutral neuroprotective effect. However, only 36% of the patients received aspirin therapy in the 2-year study of linagliptin.^10^ The high proportion of antiplatelet medication use in our study may have neutralized the cardiovascular risk and offset potential differences between the study groups. Finally, our study had a mean of 1.17 years and a maximum of 2.83 years of follow-up, which may not have been long enough to show a beneficial effect on reducing the risk of ischemic stroke. As a result, our finding could not rule out the possible neuroprotective effect with longer-term treatment of sitagliptin.

On account of the controversy over the cardiovascular effects of sitagliptin, the trial evaluating cardiovascular outcomes with sitagliptin (TECOS), a double-blind, randomized trial, is designed for further evaluation of the safety of this medication.^29^ This trial enrolled patients with established cardiovascular diseases; however, it did not specifically include individuals with recent ischemic stroke. In our study, all the patients had recent ischemic stroke. As a result, our study currently provides the only evidence on the cerebrovascular outcome of sitagliptin treatment for T2DM in a recent ischemic stroke population.

This study has several limitations. First, the claims database did not include personal information on tobacco use, physical activity, body mass index, family history of cardiovascular disease, or laboratory parameters including glycated hemoglobin levels. Nonetheless, we were able to include a wide range of variables related to outcomes to make our 2 study groups well balanced. Second, our study is based on the assumption that patients properly adhered to instructions to use their treatment medications in the claims data. Finally, our study has a mean of 1.17 years and a maximum of 2.83 years of follow-up because sitagliptin was available in Taiwan only after March 1, 2009. Studies with longer duration of follow-up may be needed to generate more information.

In conclusion, sitagliptin use in T2DM patients who had recent ischemic stroke was not associated with increased or decreased risks of the composite adverse cardiovascular outcome, which included recurrent ischemic stroke, cardiovascular death, or MI. Sitagliptin neither increased nor reduced the risk of hemorrhagic stroke, death from any cause, or heart failure hospitalization. These findings could help clinicians in formulating strategies for use of antihyperglycemic agents in this high-risk population of patients.
